# Growth, Physiological, and Biochemical Responses of Ethiopian Red Pepper (*Capsicum annum* L.) Cultivars to Drought Stress

**DOI:** 10.1155/2023/4374318

**Published:** 2023-01-07

**Authors:** Wubetie A. Wassie, Animut M. Andualem, Abiyu E. Molla, Zelalem G. Tarekegn, Mersha W. Aragaw, Misganaw T. Ayana

**Affiliations:** ^1^Department of Biology, College of Science, Bahir Dar University, P.O. Box 79, Bahir Dar, Ethiopia; ^2^Department of Biology, College of Natural and Computational Sciences, University of Gondar, P.O. Box 196, Gondar, Ethiopia; ^3^Department of Chemistry, College of Science, Bahir Dar University, P.O. Box 79, Bahir Dar, Ethiopia

## Abstract

Red pepper (*Capsicum annum* L.) is an increasingly important economic crop in the world. Thus, this study aimed to investigate the growth, physiological, and biochemical responses of red pepper cultivars under drought stress conditions. A pot culture experiment was conducted in a completely randomized design with three replications, four treatments, and three cultivars. Totally, 36 pots and six seeds per pot were used to grow the seeds. After five weeks, the cultivars were exposed to different drought stress conditions (100% FC or control, 80% FC or low stress, 60% FC or moderate stress, and 40% FC or severe stress). All the collected data were subjected to an analysis of variance (ANOVA). Shoot length was reduced significantly (*p* < 0.05) in the Hagerew cultivar under severe drought stress. The photosynthesis rate was reduced by 21.11% (*p* < 0.05) in the Mitmita cultivar under severe drought stress. The highest percentage reduction of chlorophyll content (77.28%) was recorded in the Hagerew cultivar. Both Markofana and Mitmita responded to drought stress by increasing the accumulation of proline and phenolic compounds. The root-to-shoot ratio was increased significantly in both Markofana and Mitmita cultivars (27.91% and 50.92%), respectively, under drought-stress conditions. This study depicted that the cultivar Mitmita was the most drought-tolerant cultivar among the three cultivars.

## 1. Introduction

Red pepper (*Capsicum annum* L.) belongs to the family Solanaceae and originated in the world's tropics and subtropics over 2000 years ago [1]. The crop is grown widely under various environmental and climatic conditions. Hot pepper is the world's third most important vegetable, next to potatoes and tomatoes [2, 3], which produced approximately 40 in 2020 (World) and 7.70 (Africa) million tons of green fruit [4]. Red pepper cultivars are widely grown in various parts of Ethiopia, particularly in the Amhara, Oromia, and Southern Nations Nationalities, and Peoples Regional States regions [5]. According to MoARD [6]; the total pepper production was 0.25 million tons from 118, 987 hectares in Ethiopia. The production in the green form was 220, 791 tons from 97, 712 ha, and 118, 514 tons in the dry form from an area of 300,000 ha [7]. Peppers are important cash crops for smallholder farmers in countries like Ethiopia [8] and are good sources of nutrients in the human diet [9, 10]. However, their productivities are less since they are susceptible to horticultural plant drought stress [11–13].

The economies of most developing countries are based on agriculture, which is fully dependent on nature [14]. However, this agriculture has been affected by different biotic and abiotic factors such as drought, salinity, heat, and waterlogging stress [15, 16]. Among these, drought stress is an important issue that greatly affects agriculture productivity [17]. The insufficiency of water caused by erratic and poorly distributed rainfall causes tremendous global losses in agriculture [18]. Drought decreases the productivity and quality of crops [19] and also limits the successful utilization of land throughout the world [20]. The metabolism activities such as physiological processes become highly disturbed in plants when exposed to drought stress [21, 22]. According to Alqudah et al. [23] and Lamaoui et al. [24], drought stress is considered the most important abiotic factor that adversely affects the yield and quality of many field crops by altering the growth, physiology, and metabolic activities of plants. In particular, it reduces plant growth by affecting various physiological and biochemical processes, such as osmotic adjustments, water relations [25, 26], and photosynthetic activity [27, 28], and consequently causes a reduction in flower production [29–31].

The best approach to cope with the adverse effects of drought stresses is screening (testing) the available red pepper cultivars for water scarcity [32]. The adaptive responses to water deficit include morphological, physiological, and biochemical changes such as changes in growth rate, stomatal conductance, photosynthesis rate, chlorophyll content, and the root-to-shoot ratio [33]. Morphological and some leaf-related characteristics were also used as indicators for the detection of drought stress in chili pepper [34]. However, drought stress on the parameters of growth, physiological, and biochemical was not conducted comprehensively on the response of red pepper cultivars in the studied area to get the red pepper cultivars tolerant to drought stress. Therefore, the objective of this research was to study the effect of drought stress on selected morphological, physiological, and biochemical parameters of red pepper cultivars so that the responses of the cultivars were evaluated against drought stress.

## 2. Materials and Methods

The research was performed with three red pepper (*Capsicum annum* L.) cultivars in response to drought stress, namely, Hagerew, Markofana, and Mitmita. The seeds of these cultivars were obtained from the Bahir Dar Agricultural Research Centre. The experiment was carried out at the Botany Laboratory of the University of Gondar (12°35′ 11.7″ N and 37°26′ 27″ E, 2148m above sea level). The annual average of the maximum and minimum temperature lies approximately 27°C and 16°C, respectively, while mean relative humidity and precipitation are approximately 56% and 1161 mm, respectively. The annual wind speed and pressure for the area were found to be 7.1 km/h and 1023 mbar, respectively. During the experimental period (March to April), the relative humidity was 50.5%, the maximum and minimum daily temperatures were found to be 29.1°C and 18°C, respectively, and no rainfall occurred. The red pepper crops have a growing period of 120–210 days [35], and these three cultivars have approximately the same development periods.

Thereafter, the seeds of the three red pepper cultivars (*Capsicum annum* L.) were sterilized with ethanol (80%) for around 15 min, bathed with distilled water, and then sown in plastic pots (25 cm wide × 26 cm height) containing 6 kg of farmyard manure and soil in a 1 : 3 ratio (25% FYM and 75% soil). The soil texture was determined using the hydrometer method and it was identified as sandy loam soil (Table 1). The physical and chemical properties of the soil sample used to grow the pepper plants were analyzed as follows.

**Figure 1:**
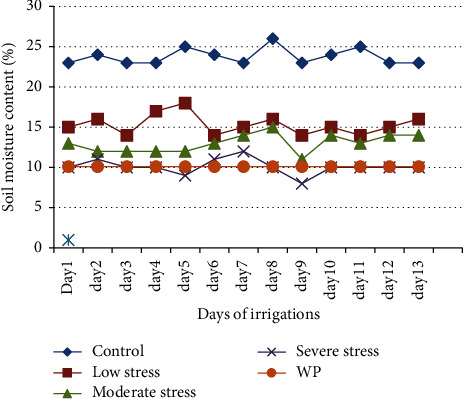
Soil moisture content depletion on different days before irrigation (WP = permanent wilting point of the soil).

Soil moisture content was determined using an instrument (IMKO, Trime-Pico TDR) in each pot under study up to the end of the experiment. Then, the moisture readings were taken directly by inserting the instrument deep into the soil after the instrument was calibrated [36]. The moisture data were recorded at regular intervals up to the end of the experiment for each treatment (Figure 1) and then irrigation was performed carefully.

**Figure 2:**
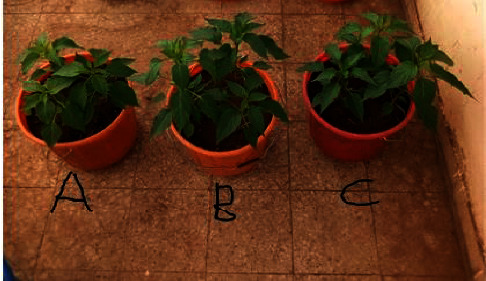
Seedlings of potted plants for the three cultivars: (a) Hagerew; (b) Markofana; (c) Mitmita.

After 2 weeks of germination, uniform-sized seedlings of the cultivar were allowed to continue in plastic pots. Therefore, each plastic pot had three seedlings up to the end of the experiment to observe the response of the cultivars against drought stress (Figures 2(a)–2(c)). The potted seedlings were watered with tap water daily at a field capacity of 100% (FC) for up to 2 weeks, which was considered the accommodation period. After 4 weeks, a completely randomized design (CRD) with three replications and four treatments was adopted for the three cultivars.

### 2.1. Drought Stress Condition

Drought condition is a feature of climate that appear when the rainfall is deficient compared to the statistical multiyear average for a region, over an extended period of a season or year, or even more [37]. To realize the drought stress, plants were subjected to the following three drought levels along with the control following the method used by Al-Maskri et al. [38]. These were low stress, or 80% FC, moderate stress, or 60% FC, severe stress, or 40% FC, and the control group, or 100% FC. For the experiment, there were 40 days of drought exposure on the seedlings for all the treatments.

### 2.2. Irrigation Water Application

The irrigation water application was done following the method used by Kazgöz Candemi̇r et al. [39]. The pots used to grow the seedlings have pores underneath for water leakage. Three pots were used where irrigation water was applied with a specific beaker at certain intervals until water began to leak from underneath the pots to determine the irrigation water amount before each irrigation. As soon as leakage was seen from underneath the pot, water application was stopped to determine the volume of water (in ml). This amount determined corresponded to the 100% FC, while 80% FC, 60% FC, and 40% FC of this amount were applied to the other pots. Accordingly, the amount of water applied per irrigation to maintain the soil field capacity was 600 ml for 100% FC, 480 ml for 80% FC, 360 ml for 60% FC, and 240 ml for 40% FC, with a scheduled irrigation interval of 3 days.

The tap water was irrigated within the three days interval up to the end of the experiment. The electrical conductivity of the tap water used in the experiment was on an average of 0.0066 dS/m, and the average pH was found to be 6.99. According to Ayers and Westcot [40]; the quality of water used in the experiment was within the permissible levels required for irrigation water.

### 2.3. Data Collection

#### 2.3.1. Growth Measurements

Shoot length, root length, number of leaves, stem thickness, number of branches, leaf area, leaf width, leaf length, and canopy diameter were measured after beginning the water treatments. Changes in the shoot and root growth of the cultivars within 10 days intervals till the end of the experiment were recorded. All the leaves and branches were counted individually in each replica at the end of the stress period. The stem thickness of each plant per pot was measured by using a Digital Vernier hand caliper meter (AP-961) and the average stem diameter of the individual plants in the replicate was taken as the stem thickness at the end of the stress period. A leaf area meter (AM 300, ADC Bio Scientific Limited, UK) was used to measure leaf area and leaf width at the end of the experiment. The canopy diameter was measured within each pot following the method of Delelegn et al. [41]. Then, the mean values of the North-South and East-West measurements were taken as canopy diameters.

#### 2.3.2. Physiological Measurements

The physiological parameters such as leaf relative water content (LRWC), chlorophyll fluorescence (CF), net photosynthesis rate (A), transpiration rate (E), and stomatal conductance (gs) were measured.

LRWC was determined from the leaves collected from the cultivars in all treatments following Kirnak et al. [42]. Similarly, sample fresh leaves were taken following the method of Moradi et al. [43] and immediately weighed using a digital electronic balance to get a fresh weight (FW). Then, the leaves were immersed in large Petri dishes containing distilled water for twenty-four (24) hours. After 24 hours, the turgid weight (TW) was determined. The leaves were then placed in a preheated oven at 72°C for 24 hours and dried to obtain their dry weight (DW). Then, the LRWC was calculated using the formula given by Kirnak et al. [42] as follows:

(1) $$\begin{array}{c} LRWC=\frac{F.W-D.W}{T.W-D.W}x100\text{,} \end{array}$$

where F.W = fresh weight; D.W = dry weight; and T.W = turgidity weight of leaves.

Chlorophyll fluorescence was measured using a portable multimode OS5P Chlorophyll Fluorometer (Opti-Sciences, Inc., USA) from 10:00 to 11:00 AM using the methods of Husen et al. [44]. Before it was recorded, the upper surface of the leaf was predarkened for 30 minutes by using leaf clips to secure a complete relaxation of all the reaction centers, as recommended by Kauser et al. [45]. PSII efficiency (*F*0/*Fm*) readings were taken from the whole plants per pot (9 leaves were taken from each plant in the pots) as recommended by Almeselmani [46]. Therefore, the maximum quantum yield of PSII efficiency (*Fv*/*Fm*) was directly taken from the instrument reading.

The net photosynthesis rate, transpiration rate, and stomatal conductance were measured at the final using a gas analyzer (LC Pro + Photometer, ADC Bio Scientific Ltd., Hoddesdon, United Kingdom). Fully expanded attached leaves were taken and all these measurements were performed on 10 leaves from each plant as recommended by Husen et al. [44].

#### 2.3.3. Biochemical Measurements

**Figure 3:**
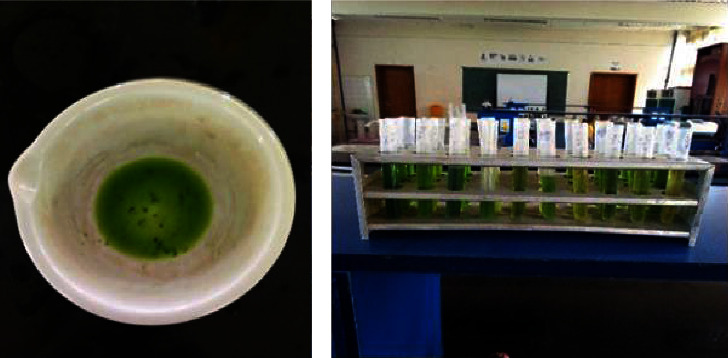
(a) Extraction and (b) determination of chlorophyll content from sample leaves.

Biochemical parameters such as chlorophyll content, internal proline content, and total phenolic compounds were determined. To determine the chlorophyll “*a*,” “*b,*” and total chlorophyll, leaf samples were taken from each plant per replica. Fresh samples and homogenization were done as shown in Figures 3(a) and 3(b). In the end, the concentration of chlorophyll “*a*” and “*b*” total chlorophyll content was determined using the formula given in Arnon [47] as follows:

  Chl. a (mgg-1FW) = 12.7 × (A663) − 2.69 × (A645)
  Chl. b (mgg-1FW) = 2.9 × (A663) − 4.68 × (A645)

Total chlorophyll concentration (mg/g FW) = 20.2 × A663 + 8.02A645.

Here (A663) and (A645) represent absorbance values read at 663 and 645 nm wavelengths. This was collected at the end of a drought stress period to compare the chlorophyll content between stressed and nonstressed red pepper plants.

Proline content was determined following the method used by Bates et al. [48] based on the reaction of proline with ninhydrin. Then, the absorbance at 520 nm was determined using a microprocessor UV-Visdouble-beam spectrophotometer. The total phenolic content of the extracts was determined following the method of Rispail et al. [49] using the following formula at the end of the experiment.

Total phenolic content = gallic acid equivalent (mg/L) × total volume of the methanol extract x sample weight (kg/g)/Dilution factor (L/mL).

#### 2.3.4. Biomass Estimation

In the end, plants were harvested carefully. Then, the shoot and root fresh weights (SFW and RFW) of each replica of the treatments for the three cultivars were measured using a digital electronic balance (CY510, Citizen Scale, Poland), and the mean values were taken as the shoot and root fresh weights of the red pepper cultivars. Shoot and root dry weights (SDW and RDW) for the cultivars were also measured using a digital electronic balance.

To get the root-to-shoot ratio of biomass, the whole plants were uprooted, rinsed, separated into shoot and root, and oven-dried for 24 hours at 72°C. Then, the root-to-shoot ratio was computed using the formula given by Luvaha et al. [50] as follows:

(2) $$\begin{array}{c} Root:Shoot=\frac{Root\;dr\;y\;weight}{Shoot\;dr\;y\;weight}X100\%\text{,} \end{array}$$

### 2.4. Data Analysis

All collected data were subjected to analysis of variance (ANOVA), mean comparisons were performed using LSD, and graphical comparisons were presented using the SPSS version 20 software. The significance level of the data was accepted at *p* < 0.05 and rejected when *p* > 0.05 confidence interval level. A one-way ANOVA was used to determine statistically significant differences between the means of the parameters of the three red pepper cultivars under different drought levels. The parameters of each of the cultivars were measured on the same independent variable after having undergone the same condition. On the other hand, a two-way ANOVA was used to analyze the interaction effect of both the cultivar type and watering regime. A correlation was also made to determine the direction of the relationship and to measure the strength of the association between two continuous variables.

## 3. Results and Discussion

### 3.1. Shoot Length

**Figure 4:**
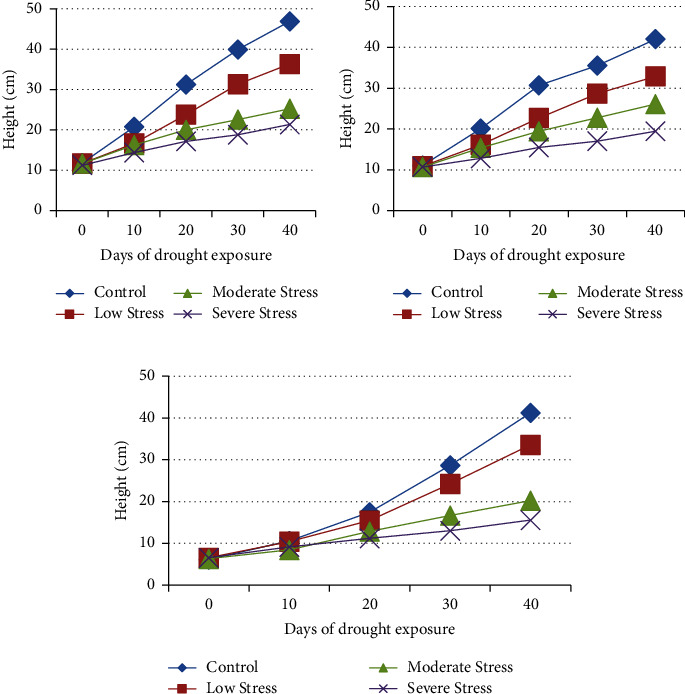
Shoot variations in (a) Hagerew, (b) Markofana, and (c) Mitmita cultivars after 40 days of drought exposure.

Changes in the shoot growth of the cultivars within 10 days intervals till the end of the experiment were recorded. The results showed that drought stress decreased the shoot length by 19.75%, 21.87%, and 31.02% at 80% FC, 60% FC, and 40% FC, respectively, in the Hagerew cultivar in the first 10 days of drought exposure compared to the control (Figure 4(a)). Similarly, the shoot length declined by 23.83%, 35.94%, and 45.19% at 80% FC, 60% FC, and 40% FC on the 20th day of drought exposure in the Hagerew cultivar. At prolonged drought exposure (40th day), shoot length declined by 21.44%, 43.46%, and 52.91% in similar treatments, and the variation was statistically significant (*F* = 39.89, *p* < 0.05).

The Markofana shoot length (SL) was also reduced by 19.91%, 23.25%, and 36.44% on the 10th day of drought exposure and by 26.08%, 36.62%, and 49.66% on the 20th day of drought exposure at 80% FC, 60% FC, and 40% FC, respectively (Figure 4(b)). At prolonged stress (40th day), the effect was statistically significant (*F* = 37.27, *p* < 0.05). Similarly, shoot length was reduced in the Mitmita crop by 1.23%, 19.90%, and 13.74% on the 10th day of drought exposure and by 11.47%, 26.09%, and 35.67% on the 20th day of drought exposure (Figure 4(c)) at 80% FC, 60% FC, and 40% FC, respectively, compared to the control. At severe drought stress (40th day), the effect was statistically significant (*F* = 45.01, *p* < 0.05). The finding in shoot reduction is in agreement with the reports on soybean [51, 52], rice [53], common bean [54], maize [55], and turfgrass [56]. Shoot length is reduced by up to 25% in citrus seedlings under drought stress conditions [57]. This is related to the reduction in cell turgor, which decreases the rate of cell division and cell expansion due to the inhibiting effect of water shortage on growth-promoting hormones [58, 59]. Another reason [60] is diminished cell expansion and a higher leaf senescence rate. According to Farooq et al. [61], the imposition of plants to drought stress disrupts water flow from the xylem to the surrounding elongating cells and causes a reduction in shoot lengths as well.

#### 3.1.1. Number of Leaves

**Figure 5:**
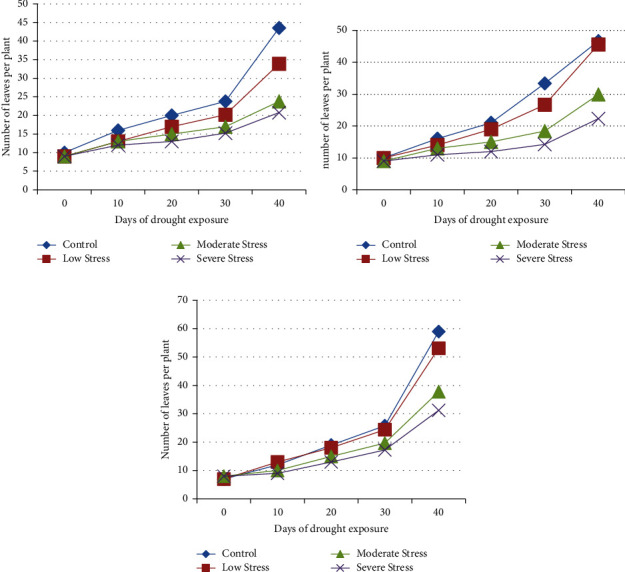
Variations in the number of leaves in the (a) Hagerew, (b) Markofana, and (c) Mitmita cultivars after 40 days of drought exposure.

It was found that the number of leaves decreased by 52.28% in the Hagerew cultivar (Figure 5(a)), by 52.15% in the Markofana cultivar (Figure 5(b)), and by 47.08% in the Mitmita cultivar (Figure 5(c)). In this study, the number of leaves decreased significantly in the Markofana cultivar at *p* < 0.05 level. This finding is similar to the previous reports by Anjum et al. [62] on plants and maize [55]. Others [63–65] also found that a water deficit could limit the growth of the plant, which can be seen by the reduced leaf number. This might be due to a reduction in leaf formation and the abscission of lower leaves. It may also be the result of leaf senescence caused by increased carbon remobilization from the leaves and their redistribution to stems and roots [66].

### 3.2. Root Length and Canopy Diameter

**Figure 6:**
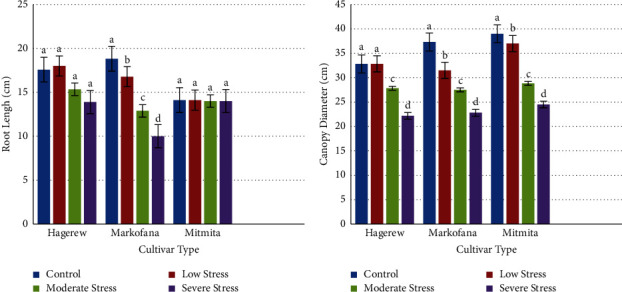
Effect of drought stress on (a) root length and (b) canopy diameter of the three cultivars. Columns with different letters are mean values ± SE of the three replicates. Bars with different letters show significant differences at *p* < 0.05 (LSD).

The root length (RL) of the three red pepper cultivars that were measured after 40 days of drought exposure is displayed in Figure 6(a). As the results showed, root length was reduced by 20.99% at 40% FC (the 40th day of drought exposure) in the Hagerew cultivar. The inhibitory effect was also significantly increased (*F* = 11.46; *p* < 0.05) in the Markofana cultivar at 40% FC as compared to the control group. The root length was reduced from 18.83 cm in the control to 10.00 cm in the 40% FC in the cultivar. On the other hand, root length declined by 27.64% in the Mitmita cultivar under severe drought stress, or 40% FC compared to the control group. Similar findings were found in marigolds [56] and mung bean plants [67]. Water deficit stress is initially sensed by the root and root growth becomes affected [68]. Similarly, different scholars reported that root length becomes impeded by drought at the early stages in alfalfa plants [69] and sunflowers [70, 71] and became shrinking in lengthened drought stress [72].

The average canopy diameter of the three red pepper cultivars was recorded from the growing plants (Figure 6(b)). Severe drought stress significantly reduced canopy diameter by 32.47%, 38.84%, and 37.18% in the Hagerew, Markofana, and Mitmita cultivars, respectively, as compared to the control. The decrease in canopy diameter was significant under severe drought stress irrespective of the control (*F* = 8.70; *p* < 0.05). This finding is in line with the work of Al-Mahmud et al. [73]; which showed that canopy diameter was significantly decreased under severe drought stress in potato plants. These might be due to the traits they inherited, and they may determine the yielding potential of the crop. The water deficits reduced the canopy growth of strawberries [65].

### 3.3. Stem Thickness and Leaf Data

The effects of drought stress on stem thickness, number of leaves, branches, leaf area, leaf width, and length of the three cultivars were recorded and presented in Table 2. The results revealed that severe drought stress or 40% FC reduced the stem thickness by 30.93% in the Hagerew cultivars compared to the control group. The stem thickness of the Markofana cultivar was also reduced by 29.33% under severe drought stress as compared to the control. On the other hand, severe drought stress decreased the stem thickness by 30.43% in the Mitmita cultivar compared to the control. The decline in stem diameter was significant in severe drought stress compared to the control. This result is in agreement with the report of Luvaha et al. [50] on mango seedlings and maize crops [55]. The result was also consistent with the previous report by Alordzinu et al. [36] on tomato plants.

The number of branches in the Hagerew cultivar was significantly affected by severe drought stress (*F* = 18.25, *p* < 0.05). Similarly, the number of branches was significantly affected under severe drought stress in both the Markofana and Mitmita cultivars (*F* = 6.11, *p* < 0.05; *F* = 17.23, *p* < 0.05), respectively. A similar finding was reported by Ichwan et al. [74] in red chili peppers under water deficit conditions. The number of branches decreased under severe drought stress in sweet peppers [75]. However, leaf width was insignificantly affected by severe drought stress in the Hagerew, Markofana, and Mitmita red pepper cultivars. The leaf length was also significantly reduced by 24.29%, 46.49%, and 41.06% under severe drought stress in the Hagerew, Markofana, and Mitmita cultivars.

The leaf area was also affected by severe drought stress in all three cultivars. As the result showed, the effect of severe drought stress on the leaf area was statistically significant (*F* = 48.76, *p* < 0.05) in the Hagerew cultivar as compared to the control. The leaf area in the Markofana cultivar was also significantly reduced (*F* = 12.13, *p* < 0.05). Similarly, the leaf area was significantly affected (*F* = 68.46, *p* < 0.05) in the Mitmita cultivar under severe drought stress. The result was consistent with the study done by Zhang et al. [76] on soybean. The reduction of leaf area under water deficit stress was also previously reported in many plants such as wheat cultivars [77], marigold plants [56], and strawberries [65, 78]. Drought stress reduced leaf area by 51.6% during the vegetative stage of cowpea due to the inhibition of cell growth [79]. According to Manandhar et al. [80], limited water availability decreases leaf area, thereby reducing plant yield. The reason for leaf growth reduction might be turgor loss and increased synthesis of abscission acid under stress [81, 82]. This is to achieve a balance between the water status of plant tissues and the water absorbed by the plant roots [83]. According to Blum [84]; a small leaf area is beneficial under drought stress to avoid hydration.

### 3.4. The Physiological Responses of Red Pepper Cultivars to Drought Stress

#### 3.4.1. Leaf Relative Water Content

Drought stress greatly reduced the physiological efficiency of leaves in the three cultivars in comparison with the controls. The degree of reduction of LRWC was high in the Hagerew cultivar (20.26%), followed by the lowest reduction of LRWC for the Mitmita cultivar (15.92%). LRWC also declined by 17.33% in the Markofana cultivar (Table 3). LRWC was significantly reduced as drought stress increased compared to control in the Hagerew cultivar. In line with this finding DaCosta and Huang [85] also found that a water deficit reduces LRWC in wheat cultivars. This was also confirmed in the previous reports [86–90]. The LRWC decreased as a result of high water stress levels and increasing resistance to water flow in the stems and leaves of plants [77, 91, 92]. However, the LRWC between the control and severe drought stress in the Markofana and Mitmita cultivars was statistically insignificant. This may be due to the variation in the ability of red pepper cultivars to avoid stress by maintaining tissue turgor osmotically. Tezera et al. [82] also found that reduced water availability in soil lowers leaf water content, causing guard cells to lose turgor, and hence the size of stomatal pores is reduced.

#### 3.4.2. Chlorophyll Fluorescence

Under severe drought stress, the percentage reduction of chlorophyll fluorescence (*Fv*/*Fm*) was 3.33% in the Hagerew, 3.70% in the Markofana, and 2.89% in the Mitmita cultivars (Table 3). In the present study, drought stress imposed for 40 days had a statistically significant effect on the PSII photochemical efficiency (*Fv*/*Fm*) of red pepper cultivars. This is in agreement with the earlier reports by Liu et al. [93] on plants, Wang et al. [94] on apple tree leaves, and Widuri et al. [34] on chili pepper plants. A significant reduction was also perceived in barley, young wheat, and pepper crop plants under drought stress [95–97]. This may be because of the suppression of PSII by decreasing electron transport and the release of magnesium and calcium ions from their binding [98, 99]. The changes in PSII activity under water deficit stress are related to photoinhibition rather than to direct damage to PSII [100]. Moreover, the reduction might be due to the development of slowly relaxing quenching processes [101].

#### 3.4.3. Photosynthetic Rate and Stomatal Conductance

The analysis of variance in the photosynthetic rate of both Hagerew and Markofana cultivars showed significant variation at *p* < 0.05 (Table 3) under severe drought conditions. However, there was an insignificant variation in the photosynthesis rate of the Mitmita cultivar. The Mitmita cultivar had the highest assimilation rate of 15.00 *μ*molem^−2^ S^−1^ followed by Markofana at 13.56 *μ*molem^−2^ S^−1^, and the lowest was noted in the Hagerew cultivar with a mean value of 12.78 *μ*molem^−2^ S^−1^. Stomatal conductance was also reduced by 4.33%, 10.97%, and 11.45% in the Mitmita, Markofana, and Hagerew cultivars, respectively, under severe drought stress conditions. In the present study, the photosynthetic rate was significantly affected under severe drought stress conditions in the Hagerew cultivar, which was consistent with the reports on sorghum [102], rice [103], and chickpea cultivars [104]. Zhang et al. [105] also proved that water stress inhibited the process of photosynthesis in maize. Drought stress decreases guard cells and causes the stomata to close, which in turn inhibits the uptake of CO_2_ needed for photosynthesis [106–108]. The stomatal conductance also decreased with increasing drought stress levels. This determines plant tolerance to drought [109]. This is because the closing of stomata to restrict gas exchange between the atmosphere and the leaf is one of the first responses of plants to drought. Mafakheri et al. [102] also reported that the decrease in photosynthesis rate can be due to both stomatal and nonstomatal factors. Related to this, Berahim et al. [110] described how stomatal movement is a critical attribute in monitoring water transpiration and CO_2_ absorption under drought stress. Stomata activity causes a change in the photosynthetic rate under drought stress conditions [111]. Chaves and Oliviera [112] also presented that stomatal conductance only affects the photosynthesis rate under severe drought stress. In this study, however, moderate drought stress also decreased the photosynthesis rate. In line with this finding, Flexas and Medrano [113] found that mild and moderate drought stresses decreased photosynthesis due to the stomatal closure and the resulting CO_2_ deficit in the chloroplasts.

#### 3.4.4. Transpiration Rate

The cultivars showed a significant difference in transpiration rates at *p* < 0.05 (Table 3). The Mitmita red pepper cultivar had a significantly higher transpiration rate than the other cultivars. The highest transpiration rate was observed in the Mitmita cultivar with a mean value of 4.99 mmol m^−2^ S^−1^ under severe drought conditions. Relatively, the Hagerew cultivar showed the lowest transpiration rate (3.23 mmol m^−2^ S^−1^) followed by the Markofana cultivar (4.11 mmol m^−2^ S^−1^). Table 3 shows that the higher the drought stress level (40% FC) is, the lower the transpiration rate of all red pepper cultivars. This finding agrees with the previous reports on wheat crops [114]. A significant reduction in the transpiration rate was also observed under drought stress conditions in crops such as wheat, rice, and maize [115, 116].

### 3.5. The Biochemical Responses of Red Pepper Cultivars to Drought Stress

#### 3.5.1. Chlorophyll Content

The leaf pigments (chlorophylls “*a*,” “*b*”, and total chlorophyll) were quantified and the results indicated that the chlorophyll content declined with an increase in the drought stress level (Table 4). Under severe drought stress, chlorophyll “*a*” was reduced by 72.14%, chlorophyll “*b*” by 28.49%, and total chlorophyll by 77.28% in the Hagerew cultivar. In the Markofana cultivar, chlorophyll “*a*” was reduced by 57%, chlorophyll “*b*” by 23.33%, and total chlorophyll by 45.19% at 40% FC. Similarly, in the Mitmita cultivar chlorophyll “*a*” was reduced by 52.35%, chlorophyll “*b*” by 18%, and total chlorophyll by 45.45% under severe drought stress or at 40% FC compared to the control group (Table 4). This reduction in pigment content was previously reported in several crop plants, including bread wheat [117], sorghum [3], millet [118], red chili varieties [119], and different sunflower varieties [70]. Others [65, 120, 121] also found that lower water field capacity tends to decrease total chlorophyll in chamomile, strawberries, and peanut plants, respectively. The reduction may be because of the low activity of the photosynthetic elements under stress. It may also be because of the reduced synthesis of the main chlorophyll pigment complexes encoded by the Cab gene family due to drought stress [122]. Chloroplasts are destructed by the reactive oxygen species under drought stress [123–125]. Chiral macroaggregates of light-harvesting chlorophyll “*a*” or “*b*” pigment-protein complexes may also be destructed [126]. On the other hand, there were insignificant differences in the treatments of both the Markofana and the Mitmita cultivars compared to the control. This may be due to the production of osmolytes during stress in the cultivars. Similarly, Ichwan et al. [74] reported that red chili pepper increases osmolytes as the mechanism of drought stress tolerance.

### 3.6. Proline Content and Total Phenolic Compound

Proline content was significantly increased under water deficit stress in the Markofana cultivar (Table 5). However, the increase in proline content was not significantly affected by the stress in the Hagerew cultivar. Proline content increased by 37%, and by 22.14% in the Hagerew and Markofana cultivars, respectively. Similarly, drought stress also influenced the total leaf phenolic content (*p* < 0.05) in the cultivars (Table 5). Under severe drought stress, total phenolic content increased by 22.57%, 44.12%, and 47.11% in the Hagerew, Markofana, and Mitmita cultivars, respectively. Crops respond to drought stress by accumulating osmolytes in response to stress [127–129]. In the present study, there was a significant increase in the proline content in the Markofana and Mitmita cultivars under severe drought stress. This agrees with the previous reports on pepper plants [130]. Similar findings were also previously reported on pea cultivars [131], Petunia hybrida [132], and soybean genotypes [133–135] under drought stress. Ghorbanli et al. [136] and Choudhary et al. [137] also reported that an increase in proline content keeps cell water levels under drought stress. Increased proline provides osmoprotective functions during drought stress [93, 138, 139]. The higher proline content under water stress signals multiple responses as part of the adaptation process in plants [140, 141]. There was a great effect of drought stress on the total phenolic content of the three cultivars (*p* < 0.05). In the present study, the total phenolic content in the sample leaves increased significantly with increasing drought stress levels in the Markofana cultivar. This agrees with the previous reports on capsicum species [96]. The synthesis of phenolic compounds due to drought stress has also been reported in many studies [131, 142–144]. This and other phenolic compounds are highly involved in protection against drought stress [145]. According to Heim et al. [146]; phenolic compounds protect against oxidative damage to cells and increment the stability of cell membranes.

### 3.7. Biomass Estimation

#### 3.7.1. Shoot and Root Fresh Weight

**Figure 7:**
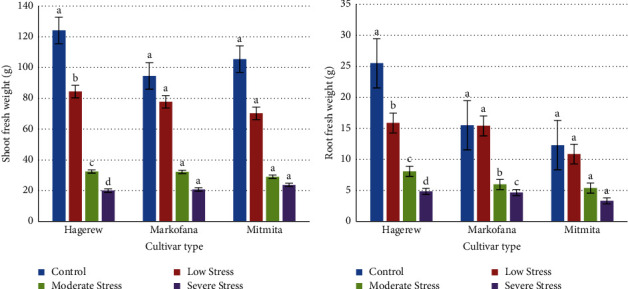
(a) Effect of drought stress on the shoot. (b) Root fresh weight of the three cultivars. Columns with different letters are the mean values ± SE of the three replicates. Bars with different letters show significant differences at *p* < 0.05 (LSD).

At the end of the experiment, all plants were collected for biomass analysis. As the results revealed, shoot fresh weight declined by 83.83%, 77.99%, and 77.50% in the Hagerew, Markofana, and Mitmita cultivars, respectively, under severe drought stress compared to the control group (Figure 7(a)). The result of this study also revealed that the highest shoot fresh weight reduction was observed in the Hagerew cultivar. Similarly, the fresh root weight of the Hagerew, Markofana, and Mitmita cultivars was reduced by 80.93%, 69.95%, and 73.09%, respectively, under severe drought stress conditions (Figure 7(b)). There was a significant reduction in dry matter (biomass) of red pepper cultivars (*p* < 0.05) at severe drought stress compared to the control group. This is in agreement with the previous reports by [42, 147–149]. This may be because of the reduction in the leaf area index that resulted in reduced photosynthesis. Another possible reason might be the close of leaves openings by signals of roots, which leads to a reduction in leaf gas exchange and ultimately lead to decreased biomass [150]. Nam et al. [151] also described that inhibition of dry matter production is largely due to the inhibitory effects of drought on leaf expansion, leaf development, and consequently reduced light interception.

### 3.8. Shoot and Root Dry Weight

Drought stress significantly affected both the shoot and root dry weights of the cultivars (Table 6). Under severe drought stress, the shoot dry weight declined by 75.29%, 83.23%, and 81.97% in the Markofana, Hagerew, and Mitmita cultivars, respectively, compared to the control. In this stress condition, both shoot and root dry biomass were significantly (*p* < 0.05) increased compared to the control in the Markofana and Mitmita cultivars. This is in agreement with the reports of Kerepesi and Galiba [152] on wheat plants. The finding also confirmed the work of Westgate and Boyer [153] and Wu and Cosgrove [154]. Leport et al. [155] and Liu et al. [93] also reported that drought stress decreases shoot and root dry weight with more influences on shoots than on roots, which increased the root-to-shoot ratio. This may be the result of increased root length rather than inhibited shoot growth under severe drought stress. It may also be due to the more extensive growth of adventitious and tap roots in plants exposed to severe water deficits than the control ones [50]. This in turn may be due to an increase in phytohormones under stress conditions than in the normal period. Chaves and Oliveria [112] also reason that higher root growth under water deficit conditions can increase drought tolerance in plants.

### 3.9. ANOVA Results

The interaction effect of watering level and cultivar type on the shoot length, leaf area, shoot fresh weight, root fresh weight, root dry weight, and total chlorophyll is presented in Table 7. As the results showed, there was a statistically significant interaction between the effects of watering level and cultivar type on the root fresh weight of the cultivars (*p*=0.003). However, there was a statistically insignificant interaction between the effects of the cultivar type and watering level on shoot length, leaf area, shoot fresh weight, total chlorophyll, and root dry weight among the three cultivars. The results also showed there was no statistically significant difference in shoot fresh weight (*p*=0.055) and total chlorophyll (*p*=0.415) between the cultivars. On the other hand, there were statistically significant differences between shoot length, leaf area, and shoot fresh weight (*p* < 0.05).

### 3.10. Correlation Results in the Three Cultivars

Correlations between various morphological and physiological parameters were made and summarized as follows presented in Tables 8–10 for Hagerew, Markofana, and Mitmita cultivars (*p* < 0.01, or *p* < 0.05 level). As the result revealed the most correlated parameters were shoot fresh weight and shoot dry weight in Hagerew (*r* = 0.985, *p* < 0.01), in Markofana (*r* = 0.997, *p* < 0.01), and in Mitmita (*r* = 0.989, *p* < 0.05). This indicates that an increase in shoot fresh weight increases the shoot dry weight in the cultivars. On the other hand, the least correlated parameters were root length with shoot fresh weight (*r* = 0.754) at the 0.01 level in the Hagerew cultivar. Root fresh weight with shoot length is the least correlated parameter in both Markofana (*r* = 0.873) and Mitmita (*r* = 0.753) at a 0.01 level.

## 4. Conclusion

Based on the results, it can be concluded that cultivar Mitmita was the most drought tolerant among the three cultivars. This was manifested by insignificant reduction values of root length, shoot length, stem thickness, leaf width, leaf relative water content, chlorophyll fluorescence, photosynthesis rate, transpiration rate, chlorophylls “*a*,” “b”, and total chlorophylls, shoot fresh weight, and root fresh weight. The cultivar also had a higher accumulation of biochemical metabolites, mainly proline and total phenolic compounds, against drought stress. To fully utilize the potential of the studied cultivar Mitmita, further studies on molecular and biochemical drought response mechanisms of the cultivars and agronomic and nutritional evaluation experiments are recommended.

## Figures and Tables

**Table 1:** Physical and chemical properties of the experimental soil.

Properties examined	Units	Values
Sand	%	62.57
Silt	%	22.68
Clay	%	14.75
Moisture saturation	%	30.4
Wilting point value	%	10.1
Field capacity moisture	%	24.5

Textural classification		Sandy loam soil
Electrical conductivity	ms/cm	0.68
pH		7.21
OM	%	0.22

**Table 2:** Morphological responses of the cultivars against drought stress conditions.

Cultivars	Treatments	Stem thickness (mm)	Number of branches (per plant)	Leaf area (mm^2^)	Leaf width (mm)	Leaf length (mm)
Hagerew	Control	5.27 ± 0.38^a^	3.22 ± 0.48^a^	9688.67 ± 232.54^a^	56.2 ± 17.38^a^	156.57 ± 8.69^a^
	Low stress	4.89 ± 0.20^a^ (7.21)	2.33 ± 0.00^a^ (27.64)	9425.33 ± 458.83^a^ (2.72)	55.13 ± 5.95^a^ (1.90)	199.70 ± 10.72^b^ (27.55)
	Moderate stress	3.96 ± 0.22^b^ (30.93)	0.67 ± 0.38^b^ (79.19)	7011.67 ± 185.93^b^ (27.63)	46.97 ± 2.43^a^ (16.42)	157.5 ± 2.06^a^ (0.59)
	Severe stress	3.64 ± 0.23^c^ (30.93)	0.22 ± 0.22^c^ (93.17)	4235.67 ± 479.79^c^ (56.28)	36.33 ± 4.08^a^ (35.36)	118.53 ± 7.39^c^ (24.29)

Markofana	Control	4.91 ± 0.21^a^	4.67 ± 1.35^a^	10645.67 ± 1473.06^a^	102.10 ± 29.85^a^	226.30 ± 37.79^a^
	Low stress	4.73 ± 0.13^a^ (3.67)	5.33 ± 0.58^a^ (14.13)	7601.33 ± 201.85^b^ (28.59)	51.06 ± 2.32^a^ (49.99)	163.63 ± 2.67^a^ (27.69)
	Moderate stress	3.70 ± 0.11^b^ (21.77)	2.44 ± 0.56^a^ (47.75)	5319.00 ± 672.50^c^ (50.04)	40.20 ± 2.55^a^ (60.63)	139.20 ± 11.68^b^ (38.49)
	Severe stress	3.47 ± 0.24^c^ (29.33)	1.34 ± 0.22^b^ (76.23)	4151.00 ± 215.71^d^ (61.01)	33.53 ± 6.23^a^ (67.16)	121.10 ± 4.97^c^ (46.49)

Mitmita	Control	4.83 ± 1.01^a^	7.22 ± 0.45^a^	8242.67 ± 380.37^a^	47.43 ± 5.25^a^	181.67 ± 15.33^a^
	Low stress	4.69 ± 0.32^a^ (2.89)	6.55 ± 0.22^a^ (9.28)	5479.67 ± 217.29^b^ (33.52)	29.03 ± 5.95^a^ (39.43)	152.33 ± 1.59^b^ (16.15)
	Moderate stress	3.66 ± 0.06^a^ (24.22)	3.00 ± 0.51^b^ (58.45)	4150.33 ± 277.49^c^ (49.65)	31.43 ± 2.18^a^ (33.73)	130.50 ± 1.53^c^ (28.17)
	Severe stress	36 ± 0.03^a^ (30.43)	3.11 ± 0.80^c^ (56.93)	2853.33 ± 203.98^d^ (65.38)	29.97 ± 1.35^a^ (36.81)	107.07 ± 4.10^d^ (41.06)

The values represent the mean ± SE of the three replicates. Numbers followed by different letters in the columns indicate significant differences (*p* < 0.05) according to the LSD test values within parenthesis are percent variations as obtained from the control plants of respective cultivars.

**Table 3:** Physiological responses of the three red pepper cultivars against drought stress.

Cultivar	Treatments	LRWC (%)	CF (*Fv*/*Fm*)	*A* (*μ* mol CO_2_ m^−2^s^−1^)	*E* (m mol m^−2^s^−1^)	Gs (mol m^−2^ s^−1^)
Hagerew	Control	83.86 ± 1.81^a^	0.7533 ± 0.02^a^	16.87 ± 0.74^a^	5.11 ± 0.19^a^	0.199 ± 0.007^a^
	Low stress	82.26 ± 3.37^a^ (1.92)	0.6823 ± 0.02^a^ (1.59)	15.71 ± 0.66^b^ (20.70)	5.27 ± 0.20^b^ (3.33)	0.188 ± 0.004^b^ (1.11)
	Moderate stress	77.77 ± 3.38^a^ (7.26)	0.6690 ± 0.003^a^ (3.50)	14.27 ± 0.34^bc^ (23.56)	4.67 ± 0.13^c^ (4.35)	0.182 ± 0.002^c^ (2.55)
	Severe stress	66.87 ± 6.19^b^ (20.26)	0.6702 ± 0.009^b^ (3.33)	12.78 ± 0.53^d^ (26.78)	3.23 ± 0.11^d^ (5.45)	0.111 ± 0.001^d^ (11.45)

Markofana	Control	86.44 ± 3.01^a^	0.7630 ± 0.03^a^	15.79 ± 0.84^a^	5.81 ± 0.31^a^	0.185 ± 0.005^a^
	Low stress	85.14 ± 3.45^a^ (1.50)	0.6533 ± 0.03^a^ (4.35)	15.32 ± 0.54^b^ (21.45)	5.23 ± 0.29^a^ (3.35)	0.188 ± 0.006^b^ (1.08)
	Moderate stress	78.08 ± 2.36^a^ (9.67)	0.6423 ± 0.02^a^ (5.96)	14.35 ± 0.44^c^ (22.21)	4.97 ± 0.25^a^ (4.11)	0.167 ± 0.004^c^ (3.83)
	Severe stress	71.46 ± 14.21^a^ (17.33)	0.6577 ± 0.006^a^ (3.70)	13.56 ± 0.47^d^ (24.35)	4.11 ± 0.16^a^ (5.00)	0.134 ± 0.003^d^ (10.97)

Mitmita	Control	87.41 ± 1.67^a^	0.7713 ± 0.008^a^	17.12 ± 0.89^a^	6.76 ± 0.45^a^	0.198 ± 0.007^a^
	Low stress	86.57 ± 3.01^a^ (0.96)	0.7030 ± 0.007^a^ (0.24)	17.00 ± 0.79^a^ (10.11)	6.45 ± 0.40^a^ (3.23)	0.199 ± 0.007^a^ (1.02)
	Moderate stress	82.44 ± 0.54^a^ (5.69)	0.6707 ± 0.02^a^ (4.36)	16.34 ± 0.47^a^ (19.99)	5.67 ± 0.32^a^ (3.56)	0.178 ± 0.005^a^ (2.66)
	Severe stress	73.49 ± 0.003^a^ (15.92)	0.6810 ± 0.01^a^ (2.89)	15.00 ± 0.74^a^ (21.11)	4.99 ± 0.22^a^ (3.00)	0.166 ± 0.003^a^ (4.33)

The values represent the mean ± SE of the three replicates. Numbers followed by different letters in the columns indicate significant differences (*p* < 0.05) according to the LSD test values within parenthesis are percent variations as obtained from the control plants of respective cultivars.

**Table 4:** Effects of drought stress on chlorophyll content.

Treatments	Chlorophyll “*a*” (mgg-1FW)			Chlorophyll “*b*” (mgg-1FW)			Total chlorophyll (mgg-1FW)		
	Hagerew	Markofana	Mitmita	Hagerew	Markofana	Mitmita	Hagerew	Markofana	Mitmita
Control	23.80 ± 3.05^a^	16.21 ± 3.19^a^	17.61 ± 1.69^a^	1.79 ± 0.63^a^	1.80 ± 0.83^a^	3.00 ± 0.18^a^	49.61 ± 8.30^a^	33.92 ± 5.57^a^	31.09 ± 3.85^a^
Low stress	16.59 ± 6.23^a^ (52.66)	7.60 ± 1.91^a^ (48.89)	10.62 ± 3.65^a^ (39.69)	4.85 ± 3.43^b^ (1.71)	1.85 ± 0.56^a^ (2.78)	2.61 ± 0.79^a^ (0.13)	30.78 ± 13.59^a^ (37.96)	20.49 ± 2.92^b^ (39.59)	18.89 ± 6.37^a^ (39.24)
Moderate stress	10.78 ± 5.07^a^ (54.71)	7.71 ± 4.58^a^ (52.44)	11.01 ± 4.43^a^ (37.48)	1.61 ± 0.82^c^ (10.06)	2.16 ± 1.05^a^ (20.00)	2.74 ± 0.63^b^ (8.67)	19.74 ± 9.60^a^ (60.21)	19.18 ± 7.35^a^ (43.45)	22.44 ± 10.11^a^ (27.82)
Severe stress	6.63 ± 5.47^a^ (72.14)	6.97 ± 1.91^a^ (57.00)	8.39 ± 2.92^a^ (52.35)	1.28 ± 0.75^d^ (28.49)	2.22 ± 0.66^a^ (23.33)	2.46 ± 0.73^a^ (18.00)	11.27 ± 4.96^b^ (77.28)	18.59 ± 2.47^a^ (45.19)	16.96 ± 5.98^a^ (45.45)

The values represent the mean ± SE of the three replicates. Numbers followed by different letters in the columns indicate significant differences (*p* < 0.05) according to the LSD test values within parenthesis are percent variations as obtained from the control plants of respective cultivars.

**Table 5:** Effects of drought stress on proline and total phenolic content after 40 days of drought exposure.

Treatments	Proline content (*μ*mol/g)			Total phenolic (mg/100 g)		
	Hagerew	Markofana	Mitmita	Hagerew	Markofana	Mitmita
Control	3.80 ± 0.25^a^	4.21 ± 0.69^a^	4.89 ± 0.78^a^	19.79 ± 1.63^a^	21.61 ± 1.83^a^	22.34 ± 1.98^a^
Low stress	4.59 ± 0.26^a^ (12.66)	5.60 ± 1.91^b^ (18.89)	4.99 ± 0.80^b^ (6.9)	27.85 ± 4.01^a^ (14.89)	29.85 ± 1.56^b^ (13.59)	30.45 ± 1.83^b^ (15.67)
Moderate stress	5.78 ± 0.07^a^ (14.71)	6.71 ± 4.58^c^ (19.44)	7.45 ± 0.81^c^ (21.78)	36.51 ± 0.82^a^ (19.56)	31.26 ± 0.05^c^ (28.00)	32.33 ± 0.83^c^ (29.0)
Severe stress	9.63 ± 0.47^a^ (22.14)	10.97 ± 1.91^d^ (37.00)	11.23 ± 1.92^d^ (40.00)	45.28 ± 0.75^a^ (22.57)	36.32 ± 0.66^d^ (44.12)	38.11 ± 1.83^d^ (47.11)

The data represent the mean ± SE of the three replicates in the experiment. Means followed by the different letters in a column are significantly different at *p* < 0.05 level according to the LSD test values within parenthesis are percent variation as obtained from the control plants of respective cultivars.

**Table 6:** Effects of drought stress on the biomass formation of red pepper cultivars.

Cultivars	Treatments	RDW (g)	SDW (g)	Root: shoot ratio
Hagerew	Control	3.26 ± 0.51^a^	26.72 ± 2.11^a^	12.07 ± 1.18^a^
	Low stress	2.31 ± 0.09^a^ (29.14)	15.27 ± 0.85^b^ (11.45)	15.25 ± 1.35^a^ (26.35)
	Moderate stress	1.14 ± 0.06^b^ (65.03)	6.59 ± 0.49^c^ (75.34)	17.64 ± 2.28^a^ (46.15)
	Severe stress	0.91 ± 0.29^c^ (72.09)	4.48 ± 0.38^d^ (83.23)	19.97 ± 5.57^a^ (65.45)

Markofana	Control	2.08 ± 0.13^a^	16.55 ± 0.35^a^	12.61 ± 0.91^a^
	Low stress	1.98 ± 0.12^a^ (4.81)	12.99 ± 1.83^b^ (21.51)	15.74 ± 2.16^b^ (24.82)
	Moderate stress	0.89 ± 0.09^b^ (57.21)	6.16 ± 0.28^c^ (62.78)	14.65 ± 0.77^c^ (16.18)
	Severe stress	0.66 ± 0.00^c^ (68.27)	4.04 ± 0.06^d^ (75.29)	16.13 ± 0.24^d^ (27.91)

Mitmita	Control	2.44 ± 0.32^a^	18.03 ± 0.46^a^	13.51 ± 1.74^a^
	Low stress	1.84 ± 0.24^a^ (24.59)	11.38 ± 0.75^b^ (36.88)	16.09 ± 1.59^b^ (19.09)
	Moderate stress	0.91 ± 0.12^b^ (62.70)	5.12 ± 0.58^c^ (71.60)	17.87 ± 1.46^c^ (32.27)
	Severe stress	0.51 ± 0.08^c^ (79.89)	3.25 ± 0.97^d^ (81.97)	20.39 ± 0.06^d^ (50.92)

The values represent the mean ± SE of the three replicates. Numbers followed by different letters in the columns indicate significant differences (*p* < 0.05) according to the LSD test values within parenthesis are percent variations as obtained from the control plants of respective cultivars.

**Table 7:** ANOVA results of the effect of drought stress on some parameters of the three red pepper cultivars.

Mean squares							
Sources of variation	Df	SL	LA	SFW	RFW	RDW	Total chl
Watering level (W)	3	1083.796^*∗∗*^	56266014.028^*∗∗*^	14770.054^*∗∗*^	360.722^*∗∗*^	7.188^*∗∗*^	879.703^*∗∗*^
Cultivar type (C)	2	70.919^*∗∗*^	18690886.861^*∗∗*^	292.381^ns^	95.109^*∗∗*^	0.936^*∗∗*^	148.565^ns^
WXC	6	8.373^ns^	1867180.194^ns^	182.825^ns^	25.969^*∗∗*^	0.167 ns	152.930^ns^
Error	21	3.45	4.67	4.55	2.78	0.36	3.97

ns-not significant and ^*∗∗*^-significant at *p* < 0.05.

**Table 8:** Correlation between different morphological and physiological parameters in the Hagerew cultivar.

Parameters	SL	RL	SFW	RFW	SDW	RDW	Total chl
SL	1	0.677^*∗*^	0.925^*∗∗*^	0.942^*∗∗*^	0.926^*∗∗*^	0.886^*∗∗*^	0.722^*∗∗*^
RL		1	0.754^*∗∗*^	0.651^*∗*^	0.690^*∗*^	0.598^*∗*^	0.359
SFW			1	0.977^*∗∗*^	0.985^*∗∗*^	0.955^*∗∗*^	0.615^*∗*^
RFW				1	0.978^*∗∗*^	0.962^*∗∗*^	0.670^*∗*^
SDW					1	0.943^*∗∗*^	0.655^*∗*^
RDW						1	0.547
Total chl							1

^*∗*^Correlation is significant at the 0.05 level (2-tailed). ^*∗∗*^ Correlation is significant at the 0.01 level (2-tailed).

**Table 9:** Correlation between different morphological and physiological parameters in the Markofana cultivar.

Parameters	SL	RL	SFW	RFW	SDW	RDW	Total chl
SL	1	0.889^*∗∗*^	0.922^*∗∗*^	0.873^*∗∗*^	0.932^*∗∗*^	0.895^*∗∗*^	0.895^*∗∗*^
RL		1	0.913^*∗∗*^	0.896^*∗∗*^	0.915^*∗∗*^	0.922^*∗∗*^	0.313
SFW			1	0.952^*∗∗*^	0.997^*∗∗*^	0.957^*∗∗*^	0.280
RFW				1	0.935^*∗∗*^	0.990^*∗∗*^	0.199
SDW					1	0.942^*∗∗*^	0.329
RDW						1	0.247
Total chl							1

^*∗∗*^Correlation is significant at the 0.01 level (2-tailed).

**Table 10:** Correlation between different morphological and physiological parameters in the Mitmita cultivar.

Parameters	SL	RL	SFW	RFW	SDW	RDW	Total chl.
SL	1	0.885^*∗∗*^	0.942^*∗∗*^	0.753^*∗∗*^	0.944^*∗∗*^	0.883^*∗∗*^	0.322
RL		1	0.830^*∗∗*^	0.761^*∗∗*^	0.846^*∗∗*^	0.842^*∗∗*^	0.447
SFW			1	0.806^*∗∗*^	0.989^*∗∗*^	0.914^*∗∗*^	0.346
RFW				1	0.817^*∗∗*^	0.966^*∗∗*^	0.238
SDW					1	0.926^*∗∗*^	0.398
RDW						1	0.321
Total chl							1

^*∗∗*^Correlation is significant at the 0.01 level (2-tailed).

## Data Availability

The dataset that supports the findings in the study is available from the corresponding author upon request.

## Symbols and Abbreviations

*Fo*: Minimum fluorescence
*F *_*M*_: Maximum fluorescence
*Fv*: Variable fluorescence
LSD: List significance difference
Chl. a: Chlorophyll *a*
Chl. b: Chlorophyll *b*
Total chl: Total chlorophyll
SDW: Shoot dry weight
RDW: Root dry weight
df: Degree of freedom
SL: Shoot length
LA: Leaf area
RFW: Root fresh weight
SFW: Shoot fresh weight
W: Watering level
C: Cultivar type
RL: Root length
FAO: Food and agriculture organization of the united nations
MoARD: Ministry of agriculture and rural development
FYM: Farmyard manure
FC: Field capacity
ppm: Parts per million
LRWC: Leaf relative water content
CF: Chlorophyll fluorescence
*A*: Photosynthetic rate
*E*: Transpiration rate
gs: Stomatal conductance
TW: Turgid weight
DW: Dry weight
FW: Fresh weight.
